# Resilience to audiogenic seizures is associated with p-ERK1/2 dephosphorylation in the subiculum of *Fmr1* knockout mice

**DOI:** 10.3389/fncel.2013.00046

**Published:** 2013-04-25

**Authors:** Giulia Curia, Fabio Gualtieri, Regina Bartolomeo, Riccardo Vezzali, Giuseppe Biagini

**Affiliations:** ^1^Laboratory of Experimental Epileptology, Department of Biomedical, Metabolic, and Neural Sciences, University of Modena and Reggio EmiliaModena, Italy; ^2^Department of Neurology and Neurosurgery, Montreal Neurological Institute, McGill UniversityMontreal, QC, Canada

**Keywords:** acoustic stimulus, epilepsy, extracellular signal-regulated kinase (ERK), FosB, Fragile X Syndrome, hippocampus, geniculate body, subiculum

## Abstract

Young, but not adult, fragile X mental retardation gene (*Fmr1*) knockout (KO) mice display audiogenic seizures (AGS) that can be prevented by inhibiting extracellular signal-regulated kinases 1/2 (ERK1/2) phosphorylation. In order to identify the cerebral regions involved in these phenomena, we characterized the response to AGS in *Fmr1* KO mice and wild type (WT) controls at postnatal day (P) 45 and P90. To characterize the diverse response to AGS in various cerebral regions, we evaluated the activity markers FosB/ΔFosB and phosphorylated ERK1/2 (p-ERK1/2). Wild running (100% of tested mice) followed by clonic/tonic seizures (30%) were observed in P45 *Fmr1* KO mice, but not in WT mice. In P90 *Fmr1* KO mice, wild running was only present in 25% of tested animals. Basal FosB/ΔFosB immunoreactivity was higher (*P* < 0.01 vs. WT) in the CA1 and subiculum of P45 *Fmr1* KO mice. Following the AGS test, FosB/ΔFosB expression consistently increased in most of the analyzed regions in both groups at P45, but not at P90. Interestingly, FosB/ΔFosB immunoreactivity was significantly higher in P45 *Fmr1* KO mice in the medial geniculate body (*P* < 0.05 vs. WT) and CA3 (*P* < 0.01). Neurons presenting with immunopositivity to p-ERK1/2 were more abundant in the subiculum of *Fmr1* KO mice in control condition (*P* < 0.05 vs. WT, in both age groups). In this region, p-ERK1/2-immunopositive cells significantly decreased (–75%, *P* < 0.01) in P90 *Fmr1* KO mice exposed to the AGS test, but no changes were found in P45 mice or in other brain regions. In both age groups of WT mice, p-ERK1/2-immunopositive cells increased in the subiculum after exposure to the acoustic test. Our findings illustrate that FosB/ΔFosB markers are overexpressed in the medial geniculate body and CA3 in *Fmr1* KO mice experiencing AGS, and that p-ERK1/2 is markedly decreased in the subiculum of *Fmr1* KO mice resistant to AGS induction. These findings suggest that resilience to AGS is associated with dephosphorylation of p-ERK1/2 in the subiculum of mature *Fmr1* KO mice.

## Introduction

The Fragile X Syndrome (FXS) is one of the leading causes of mental retardation (Rousseau et al., 1995; Dombrowski et al., 2002). In FXS, CGG triplet expansion in the fragile X mental retardation gene (*Fmr1*) prevents the synthesis of the fragile X mental retardation protein (FMRP) (De Rubeis and Bagni, 2010), causing anatomical and functional alterations, such as abnormal dendrite spines morphology and dysfunctions in synaptic plasticity (Zalfa et al., 2006). Individuals affected by FXS suffer from mental retardation, learning disabilities, and attention deficit. They also show behavioral problems including anxiety, autism, hyperactivity, and aggression (Hagerman, 1996). FXS patients may also respond to olfactory, tactile, visual, and auditory stimuli with hyper-reactivity and convulsions (Ferri et al., 1994; Miller et al., 1999; Berry-Kravis et al., 2010). The prevalence of epilepsy in FXS is larger than in the normal population, ranging from 14 to 50% of FXS patients (Berry-Kravis, 2002).

Epilepsy associated with FXS is generally benign, spontaneously remitting during or immediately after adolescence (Singh et al., 1999). Rolandic epileptiform potentials during hand tapping suggest neurophysiological similarities between FXS and benign childhood epilepsy with centrotemporal spikes (Musumeci et al., 1994). In absence of external stimulation, epileptic condition in FXS patients has been extensively investigated (Musumeci et al., 1988, 1991, 1999; Sabaratnam et al., 2001; Berry-Kravis et al., 2010; Gauthey et al., 2010). Seizures in FXS individuals appear after the age of 2 years with some cases of late onset. They are frequently complex partial seizures, but simple partial seizures, generalized tonic-clonic seizures, febrile convulsions, and *status epilepticus* have been observed as well. Seizure foci are commonly located in frontal or temporal lobes. Although in most of the epileptic FXS patients seizures are well controlled by antiepileptic drugs (Hagerman and Stafstrom, 2009), sometimes they may be frequent, severe and unresponsive to treatments (Sabaratnam et al., 2001; Incorpora et al., 2002). Even when very mild and well controlled pharmacologically, seizures may still be dangerous for FXS patients. Indeed, recently it has been demonstrated that in an animal model of temporal lobe epilepsy (TLE), pilocarpine-treated rats experiencing seizures show an increase in dopamine neuron activity and an increase in amphetamine-stimulated locomotor activity, suggesting that TLE-associated psychosis is probably due to abnormal hippocampal overdrive of dopamine neuron activity (Cifelli and Grace, 2012). In addition, a number of well-known genetic disorders, including FXS, but also tuberous sclerosis complex and Rett Syndrome, shares epilepsy, intellectual disability and autism (Brooks-Kayal, 2011), suggesting a possible link between epilepsy and psychiatric disorders. In recent studies it has been found an increased incidence of seizures in individuals with FXS also diagnosed with autism compared to FXS patients without autism (Garcia-Nonell et al., 2008; Berry-Kravis et al., 2010). In addition, it has been shown that in another form of mental retardation, the Down Syndrome, cognitive deficit is more pronounced in patients presenting epilepsy than in Down Syndrome patients without epilepsy (Eisermann et al., 2003). These observations suggest that early life seizures can result in cellular and molecular changes that could contribute to learning and behavioral disabilities and that, similarly to Down Syndrome, also in FXS, epilepsy may play a crucial role in worsening cognitive functions. As electroencephalographic (EEG) abnormalities have been observed also in non-epileptic FXS patients (Berry-Kravis, 2002), and short non-spreading events not associated with obvious clinical manifestations (subclinical seizures) have been demonstrated in other forms of partial epilepsy (D'Ambrosio et al., 2009), abnormal brain activity can actually be a problem not restricted to the 23% of FXS population presenting with spontaneous motor seizures. Therefore, a better understanding of epileptic activity in FXS is crucial for improving quality of life of all FXS patients.

*Fmr1* knockout (KO) mice provide a suitable animal model for studying FXS because they reproduce the FXS phenotype (The Dutch-Belgian Fragile X Consorthium, 1994). Although they have not been evaluated for epilepsy prospectively by video-EEG, the presence of age-dependent epilepsy was reported in these mice (Musumeci et al., 1999, 2000). Similarly to FXS patients, they are characterized by an anomalous reaction to sensory stimuli (Musumeci et al., 2000) and show audiogenic seizures (AGS), characterized by wild running followed by clonic, tonic-clonic, and/or tonic convulsions in response to loud sounds (Henry, 1967; Musumeci et al., 2000; Chen and Toth, 2001). Recent studies have demonstrated that inhibitors of extracellular signal-regulated kinases 1/2 (ERK1/2) phosphorylation prevent AGS induction in *Fmr1* KO mice (Osterweil et al., 2010; Michalon et al., 2012; Wang et al., 2012). In order to further investigate the role of phosphorylated ERK1/2 (p-ERK1/2) in AGS in the FXS mouse model, we considered two different age groups of *Fmr1* KO mice and compared them with age-matched wild type (WT) control animals. Both genotypes were exposed to the test for AGS induction at postnatal day (P) 45 or P90. In order to verify the effects of the testing procedure and AGS induction on neuronal networks, we also investigated the expression of the activity markers FosB/ΔFosB (Biagini et al., 2005, 2008), the ideal tool available now to investigate network activity in epileptic animals (Chen et al., 1997; Biagini et al., 2005; Madsen et al., 2006) since products of the *fosB* gene family are stable and tend to accumulate in repeatedly activated neurons (Chen et al., 1997; Kelz and Nestler, 2000). Instead, c-Fos, another tool being used for many years to track acute changes in neuronal network activity (Chen and Toth, 2001), is extremely short living making c-Fos reliability questionable in cases of recurrent neuronal synchronization. In line with the previous findings in other models of epilepsy (Biagini et al., 2005, 2008), we found that FosB/ΔFosB immunoreactivity was significantly correlated with seizure induction in P45 *Fmr1* KO mice, especially in acoustic regions (medial geniculate body) and the hippocampus proper (CA3). Interestingly, p-ERK1/2 was not significantly changed in *Fmr1* KO mice experiencing seizures, but it was instead markedly decreased in the subiculum of P90 *Fmr1* KO mice that were resistant to AGS induction. These findings suggest that the disappearance of sensitivity to AGS during development is associated with dephosphorylation of p-ERK1/2 in the subiculum.

## Materials and methods

### Animals

Male C57BL/6 WT (*n* = 22) and C57BL/6 *Fmr1* KO mice (*n* = 25) were studied at P45 (*n* = 25) or P90 (*n* = 22). Mice were housed at the animal facility of the Montreal Neurological Institute and sacrificed after the testing procedure. All the procedures were approved by the Canadian Council of Animal Care and were in accordance with the European Communities Council Directive 2010/63/EU.

### Audiogenic tests

**Figure 1:**
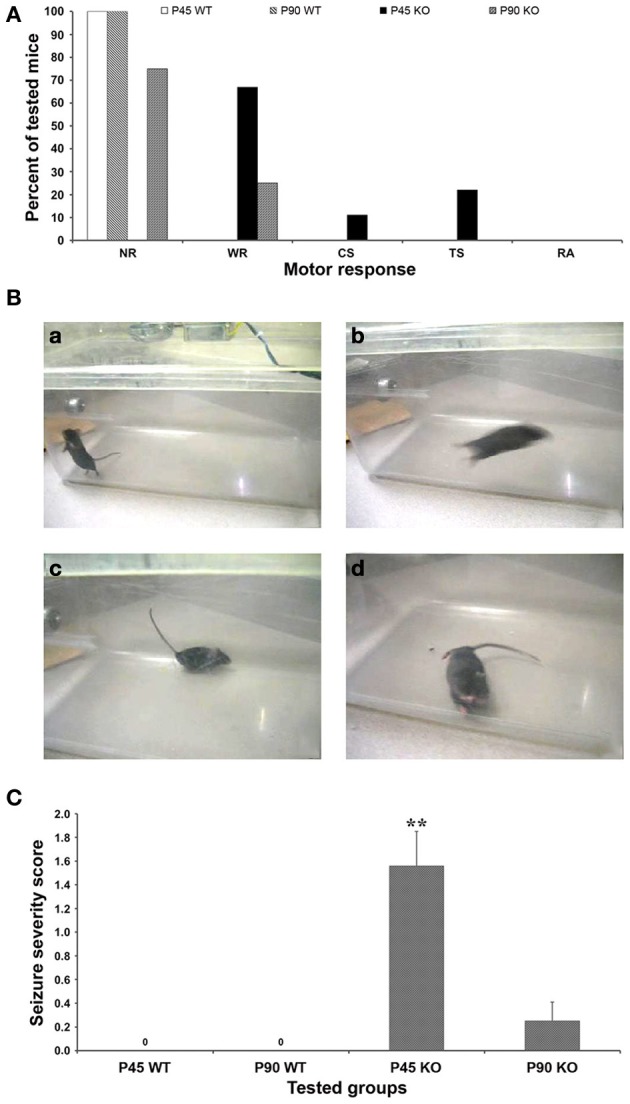
**Behavioral response to audiogenic stimuli. (A)** *Fmr1* KO mice studied at postnatal day (P) 90 showed a less intense motor response to the audiogenic stimuli (6 NR, 2 WR; *n* = 8) than P45 group (6 WR, 1 CS, 2 TS; *n* = 9), both higher than WT mice that did not respond behaviorally to the test either at P45 nor at P90. **(B)** Frames obtained from the video recordings of a P45 WT mouse during exploratory behavior (a), and of P45 *Fmr1* KO mice during WR (b), Straub tail (c), TS (d). **(C)** The seizure severity score (SSS) shows that the intensity of seizing in P45 *Fmr1* KO mice is 6-fold higher than in P90 group, both higher than WT mice (no response). ^**^P < 0.01, Kruskal–Wallis test followed by Dunn's test. NR, no response; WR, wild running; CS, clonic seizures; TS, tonic seizures; RA, respiratory arrest.

The experimental chamber consisted in a plastic cage 25 × 25 × 47 cm in which the doorbell (Electrical bell Heath Zenith, model 172C-A) was mounted on the cage roof. Mice were taken from their housing room one by one, transferred into the experimental chamber and allowed to explore the novel environment (basal noise ~65 dB) for a period of 30 s, then the bell was rung (122 dB), while concomitant behavior was video-recorded. The motor responses were classified using a scale modified from the one originally described by Jobe et al. (1973): no response (NR, in Figure 1, consisting of pause or continuous exploration), wild running (WR, in Figure 1), clonic seizure (CS, in Figure 1), tonic seizure (TS, in Figure 1), respiratory arrest and/or death (RA, in Figure 1). In order to define the intensity of the behavioral response we used the seizure severity score (SSS, in Figure 1) (Musumeci et al., 2000), consisting of a score assigned to each animal depending on its behavioral response (NR = 0, WR = 1, CS = 2, TS = 3, RA = 4). Mice of WT (*n* = 6 at P45, *n* = 4 at P90) and *Fmr1* KO (*n* = 9 at P45, *n* = 8 at P90) groups were exposed to a single 60 s continuous stimulus. As controls, WT (*n* = 6 at P45, *n* = 6 at P90) and *Fmr1* KO mice (*n* = 4 at P45, *n* = 4 at P90) were placed in the chamber but the bell was not rung.

### Immunohistochemistry and densitometric analysis

Tested WT and *Fmr1* KO mice, and untested WT and *Fmr1* KO mice were decapitated under deep isoflurane anesthesia 14–17 h after the behavioral test. Brains were extracted and cut in 1 mm-thick horizontal sections using a vibratome (VT1000S Leica, Germany); slices were fixed overnight in 4% formaldehyde, cryoprotected in 15 and 30% sucrose-buffered solutions, and then stored at −80°C. Forty μm-thick sections were obtained at the cryostat (Leica, Jung CM 3000, Germany) and processed for immunohistochemistry using a rabbit polyclonal anti-FosB/ΔFosB (H75) antibody (sc-7203, Santa Cruz Biotechnology, Santa Cruz, CA, USA; dilution 1:250) (Biagini et al., 2005, 2008) or a mouse monoclonal antibody against p-ERK1/2 (Thr202/Tyr204, cat-9106, Cell Signal Technology, Beverly, MA, USA; dilution 1:1500), according to the avidin-biotin-peroxidase complex technique and using diaminobenzidine as a chromogen (Biagini et al., 2005, 2008). Briefly, sections were treated with 3% H_2_O_2_ in phosphate buffered saline (PBS) to block endogenous peroxidase and then incubated with the primary antibody for 48 h at 4°C. Slices were then incubated with the secondary antibody (dilution 1:200) for 1 h at room temperature and finally incubated with streptavidin biotinylated horseradish peroxidase complex (dilution 1:300) for 45 min, always at room temperature.

Sections mounted on gelatin-coated slides were analyzed using the image analysis software KS300 (Zeiss-Kontron, Munich, Germany). Background values in stained sections were obtained from areas that did not contain any stained cell (i.e., the angular bundle). Stained profiles were discriminated from background throughout every sampled region (Biagini et al., 1993, 1998, 2005, 2008). Cell profile counts were determined in each field as the number of immunopositive profiles after transformation in D-circles (i.e., the diameter of circles having the same area as measured) by considering a minimum cutoff value of 7 μm. Cell counts were then divided by the sampled field area and expressed as cell densities. Sampled areas were the hippocampal regions CA1 and CA3, dentate gyrus, presubiculum, subiculum, entorhinal cortex, perirhinal cortex, lateral amygdala, primary auditory cortex, and medial geniculate body.

### Immunofluorescence

Double-immunostaining experiments were performed on free floating sections washed in PBS at room temperature and permeabilized for 1 h in PBS containing 0.1% Triton X-100 and 1% bovine serum albumin. For double immunolabeling, sections were incubated overnight in a mixture of the mouse monoclonal anti-p-ERK1/2 (1:500) and the rabbit polyclonal anti-FosB/ΔFosB antibody (1:250). Further co-labeling experiments were designed using the mouse monoclonal anti-p-ERK (1:500) and, respectively, a rabbit polyclonal antibody against parvalbumin (no. 235, Swant, Bellinzona, CH; diluted at 1:2000), neuropeptide Y (no. IHC 7180, Peninsula, San Carlos, CA, USA; diluted at 1:800), and somatostatin (no. 20089, Immunostar, Hudson, WI, USA; diluted at 1:1000). After washing, sections were incubated for 90 min at room temperature in a 1:200 dilution of goat anti-mouse AlexaFluor546® and goat anti-rabbit AlexaFluor488® (Invitrogen, Carlsbad, California). Sections were counterstained with 4′,6 diamidino-2-phenylindole (DAPI, Vector Laboratories, USA) to assess nuclear morphology. Images were visualized using a Leica TCS SP2 confocal microscope, equipped with Argon (488 nm) and Helium/Neon (543 nm).

### Statistical analysis

Data on behavioral score were analyzed with the Fisher's test. The seizure severity score was analyzed with Kruskal–Wallis non-parametric analysis of variance (ANOVA), followed by *post-hoc* Dunn's test for multiple comparisons. Cell counts underwent a Three-Way ANOVA, using as factors the genotype (WT or *Fmr1* KO), the acoustic test (yes or no) and the age (P45 or P90). *Post-hoc* test for multiple comparisons was the Fisher's Least-Significant-Difference (LSD). Data were analyzed with Sigmaplot 11 (Systat Software, San Jose, CA, USA). Results are shown as mean ± standard error of the mean (SEM), and *P* < 0.05 was considered statistically significant.

## Results

### Motor response analysis

Audiogenic stimulus was given once and concomitant animal behavior was observed and scored (Figure 1). The test for AGS did not induce anomalous behaviors in WT mice (*n* = 6 at P45 and *n* = 4 at P90; Figure 1A), which continued to explore the novel environment (Figure 1Ba) or, sometimes, paused. In contrast, a motor response was triggered in P45 *Fmr1* KO mice (*n* = 9), 6 of which developed wild running episodes (Figure 1Bb) and often presented with the Straub tail (Figure 1Bc); one progressed from wild running to clonic seizures, and 2 further progressed to tonic seizures (Figure 1Bd). In P90 *Fmr1* KO mice (*n* = 8) the response was less marked than in P45 mice: only 2 out of 8 developed wild running episodes (Figure 1A). All mice resumed normal behavior before the acoustic stimulation was over, and death due to respiratory arrest was never observed neither in P45 nor in P90 group. Motor response rate was 100% in P45 *Fmr1* KO group (9 out of 9), 25% in P90 *Fmr1* KO mice (2 out of 8) and 0% (no response) in P45 and P90 WT groups (*P* < 0.01, Fisher's test).

Using a scale created for AGS (see Materials and Methods section for details), the seizure severity score was calculated for each group: it was 1.56 ± 0.29 in P45 *Fmr1* KO mice, 0.25 ± 0.16 in P90 *Fmr1* KO mice, and 0.0 in P45 and P90 WT mice (Figure 1C). The Kruskal–Wallis test revealed a significant difference between seizure severity score at P45 and P90 *Fmr1* KO mice (*P* < 0.01). These findings confirm that FXS mice are more susceptible to AGS compared to WT mice and that this susceptibility is age-dependent, resulting more pronounced in young adult FXS mice than in older subjects (Musumeci et al., 2000, 2007; Yan et al., 2005).

### FosB/ΔFosB immunostaining

**Figure 2:**
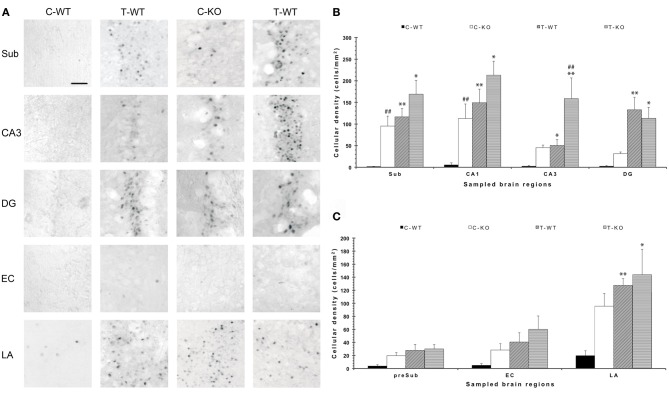
**Changes in limbic FosB/ΔFosB immunoreactivity determined by exposure to a test for audiogenic seizure induction and in relation with the mouse genotype (WT *vs*. *Fmr1* KO) at P45. (A)** Photomicrographs of FosB/ΔFosB-immunopositive cell nuclei in the Sub, CA3, DG, EC, and LA. Scale bar: 25 μm. **(B)** Bar graphs of cellular density based on counts of FosB/ΔFosB-immunopositive cell nuclei per mm^2^ in Sub, CA1, CA3 and DG. **(C)** Bar graphs of cellular density based on count of FosB/ΔFosB-immunopositive cell nuclei per mm^2^ in preSub, EC and LA. ^*^P < 0.05, ^**^P < 0.01, acoustically stimulated group vs. the respective unstimulated group; ^##^*P* < 0.01, *Fmr1* KO vs. WT mice with similar treatment (tested or not tested). Three-way ANOVA followed by LSD test. C-WT, WT mice not exposed to auditory stimulus; T-WT, WT mice exposed to the audiogenic test, C-KO, *Fmr1* KO mice not exposed to auditory stimulus; T-KO, *Fmr1* KO mice tested; Sub, subiculum; CA, cornu Ammonis; DG, dentate gyrus; EC, entorhinal cortex; LA, lateral amygdala.

**Figure 3:**
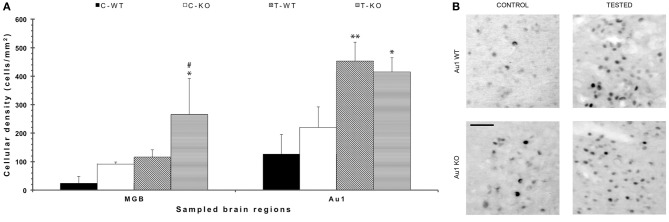
**FosB/ΔFosB immunostaining in acoustic structures after exposure to the test for audiogenic seizure induction and in relation with the mouse genotype (WT *vs*. *Fmr1* KO) at P45. (A)** Bar graphs of cellular density based on counts of FosB/ΔFosB-immunopositive cell nuclei per mm^2^ in the MGB and Au1. ^*^P < 0.05, ^**^P < 0.01, acoustically stimulated group vs. the respective unstimulated group; ^#^*P* < 0.05, *Fmr1* KO vs. WT mice with similar treatment (tested or not tested). Three-way ANOVA followed by LSD test. **(B)** Photomicrographs of FosB/ΔFosB-immunopositive cell nuclei in Au1. Scale bar: 25 μm. C-WT, WT mice not exposed to auditory stimulus; T-WT, WT mice exposed to the audiogenic test; C-KO, *Fmr1* KO mice not exposed to auditory stimulus; T-KO, *Fmr1* KO mice tested; MGB, medial geniculate body; Au1, primary acoustic cortex.

In order to disclose the neuronal networks mediating AGS, we mapped FosB/ΔFosB expression in activated neurons in hippocampal (Sub, CA1, CA3, DG in Figures 2A,B), parahippocampal (preSub, EC in Figures 2A,C) and extrahippocampal regions (LA in Figures 2A,C), including the auditory pathways (MGB, Au1 in Figures 3A,B). FosB/ΔFosB immunoreactivity was barely detectable in P45 WT mice in control condition (C-WT; Figure 2A), while it increased after the acoustic test (T-WT) in several brain regions and it reached statistical significance (*P* < 0.01, LSD test) in the subiculum, CA1, dentate gyrus, lateral amygdala (Figures 2B,C) and primary acoustic area (Figures 3A,B). A significant (*P* < 0.05) increase was present also in the CA3 hippocampal region (Figures 2A,B). Interestingly, in non-stimulated P45 *Fmr1* KO mice, FosB/ΔFosB values were significantly higher than in WT mice already in basal conditions (C-KO in Figures 2A,B), particularly in the hippocampus proper (*P* < 0.01 for CA1) and in the subiculum (*P* < 0.01; Figure 2B). Following exposure to the test for AGS, FosB/ΔFosB immunoreactivity was significantly increased in P45 *Fmr1* KO mice (T-KO in Figures 2A–C) in the hippocampus (*P* < 0.05 for CA1 and *P* < 0.01 for CA3), subiculum (*P* < 0.05), dentate gyrus (*P* < 0.05), lateral amygdala (*P* < 0.05), medial geniculate body (*P* < 0.05, Figure 3A), and primary acoustic cortex (*P* < 0.05, Figures 3A,B) compared with unstimulated *Fmr1* KO mice, while it was unchanged in the presubiculum and entorhinal cortex (Figures 2A,C). In addition, in the CA3 region the density of immunopositive neurons was significantly (*P* < 0.01) higher in P45 *Fmr1* KO mice after the test compared with age-matched WT also exposed to the testing procedure. A significant difference (*P* < 0.05) between the two genotypes, after the auditory test, was also found in the medial geniculate body (*P* < 0.05, Figure 3A), but not in other sampled areas (Figures 2B,C, 3A).

Following exposure to the test for AGS of P90 mice, FosB/ΔFosB immunoreactivity was not increased in *Fmr1* KO mice (T-KO; Table 1) compared with unstimulated P90 *Fmr1* KO mice (C-KO; Table 1) in none of the sampled areas, including acoustic regions. In addition, no differences were found in age-matched WT mice (T-WT; Table 1) also exposed to the testing procedure.

**Table 1:** **Cellular density based on count of FosB/ΔFosB-immunopositive cell nuclei per mm^2^ in *Fmr1* KO and WT mice studied at age P90**.

	**Sub**	**CA1**	**CA3**	**DG**	**preSub**	**EC**	**LA**	**MGB**	**Au1**
C-WT	117 ± 20	87 ± 40	45 ± 60	89 ± 40	25 ± 90	27 ± 60	ND	13 ± 20	47 ± 70
T-WT	67 ± 11	34 ± 21	29 ± 20	71 ± 12	25 ± 22	12 ± 50	ND	25 ± 50	32 ± 70
C-KO	76 ± 42	59 ± 37	20 ± 10	35 ± 16	12 ± 11	10 ± 70	ND	36 ± 16	33 ± 80
T-KO	79 ± 45	42 ± 20	14 ± 80	79 ± 45	14 ± 80	12 ± 10	ND	25 ± 80	78 ± 13

No significant differences were found. It was not possible to sample a sufficient number of animals for lateral amygdala (LA).

C-WT, WT mice not exposed to auditory stimulus; T-WT, WT mice exposed to the acoustic test; C-KO, Fmr1 KO mice not exposed to auditory stimulus; T-KO, Fmr1 KO mice tested; Sub, subiculum; CA, cornu Ammonis; DG, dentate gyrus; preSub, presubiculum; EC, entorhinal cortex; LA, lateral amygdala; MGB, medial geniculate body; Au1, primary acoustic area; ND, not determined.

The statistical analysis (Three-Way ANOVA) revealed main effects of test exposure in the subiculum (*P* < 0.05), CA3 (*P* < 0.05), dentate gyrus (*P* < 0.01) and amygdala (*P* < 0.05). The different genotype did not affect the level of FosB/ΔFosB immunoreactivity in any of the sampled regions, whereas age-related changes were found in the subiculum (*P* < 0.05) and CA3 (*P* < 0.05). Interestingly, a significant interaction of all the 3 main factors (genotype × test × age) was present only in the subiculum (*P* < 0.05). In the hippocampal CA1 and CA3 subfields, as well as in the subiculum, significant (*P* < 0.01 for all regions) interactions between test exposure and age, as well as between genotype and age (*P* < 0.01 for all regions) were present. Significant interactions between test exposure and age were present in the dentate gyrus (*P* < 0.01) and entorhinal cortex (*P* < 0.01); in the latter we also found a significant interaction between age and genotype (*P* < 0.05). No significant interactions were found in the amygdala. The analyzed acoustic regions presented with significant effects of test (*P* < 0.05 for the medial geniculate body; *P* < 0.01 for the primary acoustic region) and age (*P* < 0.05 for the medial geniculate body; *P* < 0.01 for the primary acoustic region), as well as significant interactions between test exposure and age (*P* < 0.05 for the medial geniculate body; *P* < 0.01 for the primary acoustic region).

### Changes of ERK1/2 activation in relation to seizures and age

**Figure 4:**
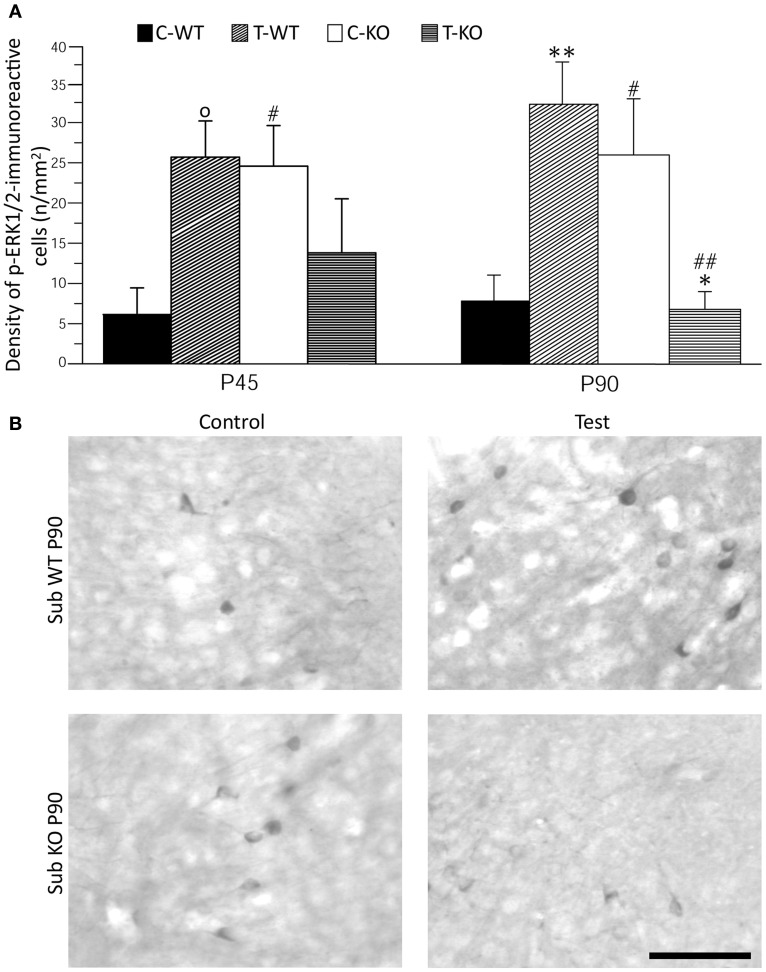
**Immunostaining of p-ERK1/2 in subiculum (Sub) after exposure to the test for audiogenic seizure induction and in relation with the mouse genotype (WT *vs*. *Fmr1* KO) and the age (P45 and P90). (A)** Bar graphs of cellular density based on counts of p-ERK1/2-immunopositive cell nuclei per mm^2^ in Sub. **(B)** Photomicrographs of p-ERK1/2-immunopositive cells in the Sub of P90 mice of both genotypes. Scale bar: 100 μm. C-WT, WT mice not exposed to auditory stimulus; T-WT, WT mice exposed to the audiogenic test; C-KO, *Fmr1* KO mice not exposed to auditory stimulus; T-KO, *Fmr1* KO mice tested. ^O^0.1 < P < 0.05, ^*^P < 0.05, ^**^P < 0.01, acoustically stimulated group vs. the respective unstimulated group; ^#^*P* < 0.05, ^##^*P* < 0.01, *Fmr1* KO vs. WT mice with similar treatment (tested or not tested). Three-way ANOVA followed by LSD test.

**Figure 5:**
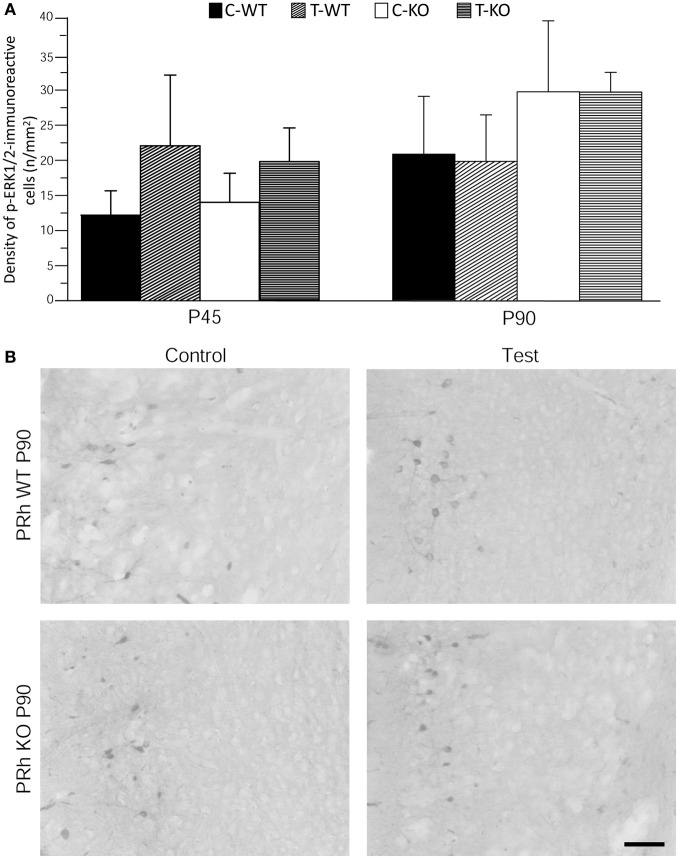
**Immunostaining of p-ERK1/2 in perirhinal cortex (PRh) after exposure to the test for audiogenic seizure induction and in relation with the mouse genotype (WT *vs. Fmr1* KO) and the age (P45 and P90). (A)** Bar graphs of cellular density based on counts of p-ERK1/2-immunopositive cell nuclei per mm^2^ in PRh. **(B)** Photomicrographs of p-ERK1/2-immunopositive cells in the PRh of P90 mice of both genotypes. Scale bar: 100 μm. C-WT, WT mice not exposed to auditory stimulus; T-WT, WT mice exposed to the audiogenic test; C-KO, *Fmr1* KO mice not exposed to auditory stimulus; T-KO, *Fmr1* KO mice tested.

As further marker of neuronal activation, we evaluated p-ERK1/2 immunoreactivity in basal conditions and after the test (Figures 4 and 5). In limbic structures, p-ERK1/2-immunoreactive cells were consistently observed in the subiculum and perirhinal cortex of both strains. Sparse immunopositive cells were also present in other regions in few mice. Thus, we focused on the subiculum and perirhinal cortex to analyze changes in p-ERK1/2-immunoreactive cell counts due to test exposure. In the subiculum, p-ERK1/2-immunoreactive cells were located in the pyramidal cell layer, as in the case of FosB/ΔFosB-immunopositive cells (cf. Figure 2A). The Three-way ANOVA revealed a significant (*P* < 0.05) effect of age and a significant (*P* < 0.01) interaction of genotype and test. Notably, a significant (*P* < 0.01) interaction among genotype, age and test was also present. Interestingly, a significant large number of p-ERK1/2-immunopositive cells was already detectable in basal conditions in *Fmr1* KO (Figure 4B), both in P45 and P90 groups of age (*P* < 0.05, Fisher's LSD test; Figure 4A). After exposure to the AGS test, p-ERK1/2-immunoreactive cells did not change significantly at P45 in both genotypes (but a trend toward increase was present in P45 WT mice; *P* = 0.06 vs. basal values), whereas a remarkable decrease (−75%) was observed in *Fmr1* KO mice at P90 (*P* < 0.05, Figure 4). This change was at variance with the significant (*P* < 0.01 vs. basal levels in WT) increase in p-ERK1/2 levels observed in WT rats exposed to AGS induction. Thus, at P90, after exposure to the AGS test, levels of p-ERK1/2-immunopositive cells in *Fmr1* KO mice were significantly less than in tested WT (*P* < 0.01; Figure 4).

Neuronal immunolabelling with the anti-p-ERK1/2 antibody was consistently observed also in the superficial layers of perirhinal cortex. We analyzed the pattern of p-ERK1/2 immunoreactivity and found them to be similar both in P45 and P90 groups and independent of exposure to the AGS test (Figure 5).

### Characterization of p-ERK1/2-immunopositive cells

**Figure 6:**
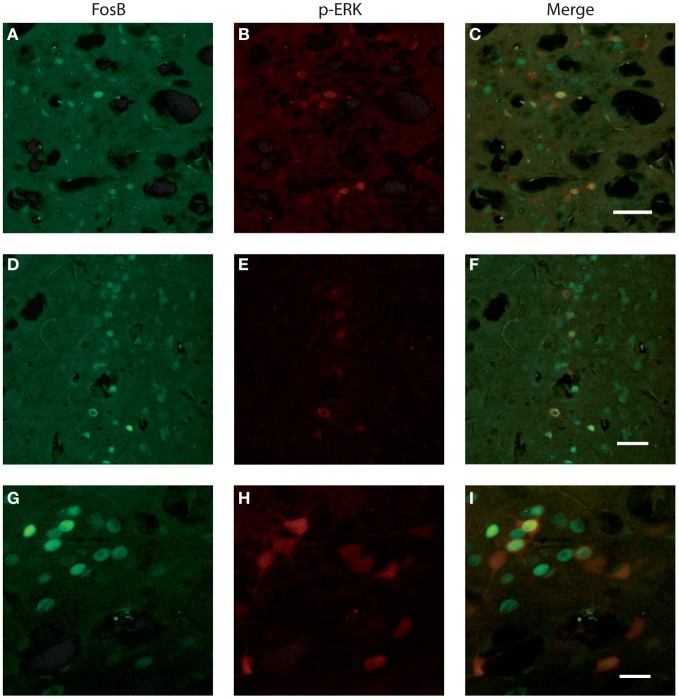
**Photomicrographs illustrating double immunofluorescence experiments with antibodies against p-ERK1/2 and FosB/ΔFosB in three exampled mice. (A–C)** illustrate the co-labeling of subicular neurons in a *Fmr1* KO mouse at P45. **(D–F)** demonstrate the co-labeling found in the perirhinal cortex of a WT mouse at P45. **(G–I)** Neurons co-labeled in the perirhinal cortex of a WT mouse showed at higher magnification. Scale bars, 50 μm for **(A–F)**, 25 μm for **(G–I)**.

We investigated whether neurons identified by FosB/ΔFosB and p-ERK1/2 antibodies were the same cells or, instead, distinct elements. Experiments of co-labeling with FosB/ΔFosB and p-ERK1/2 antibodies in P45 mice revealed that approximately 50% of p-ERK1/2-immunopositive cells expressed FosB/ΔFosB antigens, whereas the other 50% was composed by distinct neuronal elements, both in subiculum (Figures 6A–C) and perirhinal cortex (Figures 6D–I), independently of the genotype.

**Figure 7:**
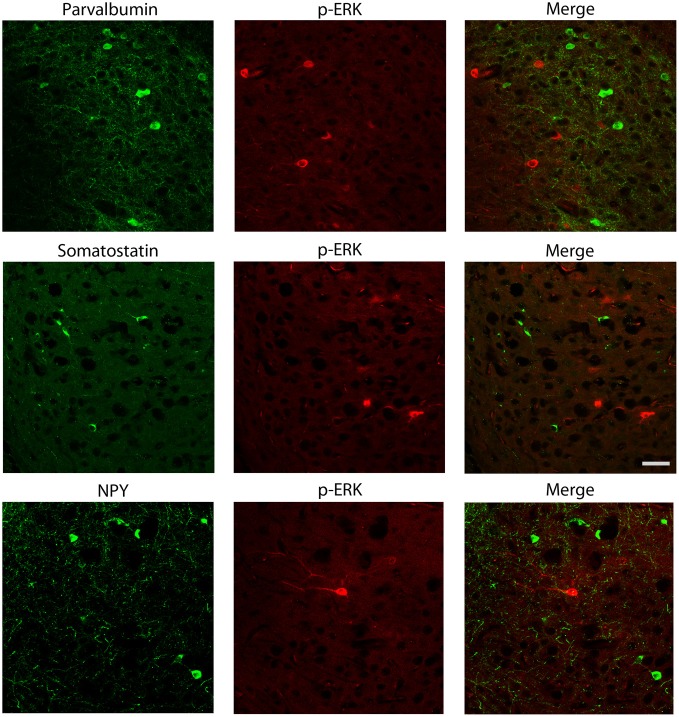
**Photomicrographs illustrating double immunofluorescence experiments with antibodies against p-ERK1/2 and markers of interneurons.** In particular, antibodies to parvalbumin (**top** panels), somatostatin (**middle** panels) or neuropeptide Y (NPY; **bottom** panels) did not co-localize with p-ERK1/2-immunopositive cells. Scale bar: 25 μm.

We also explored the possibility that p-ERK1/2-immunopositive cells could be interneurons. Experiments of co-labeling with p-ERK1/2 and markers for specific interneuron subclasses were performed in the subiculum (Figure 7) and perirhinal cortex (not shown). These experiments demonstrated that parvalbumin, neuropeptide Y and somatostatin antibodies, respectively, did not co-localize with cells stained with p-ERK1/2 antibodies (Figure 7).

## Discussion

We investigated the response to the AGS test at two different ages in mice characterized by the *Fmr1* KO genotype, compared with age-matched control WT mice. As previously reported, FXS patients (Ferri et al., 1994; Miller et al., 1999) and mice (Musumeci et al., 2000) show anomalous reaction to sensory stimuli and our experiments confirm the hyper-reaction of *Fmr1* KO mice to loud acoustic stimulus (~122 dB), given using a doorbell mounted inside the experimental cage. This experimental design gave us the possibility to test either the reaction to sensory stimuli, either the susceptibility to AGS. Reportedly, seizure susceptibility in the FXS is age-dependent: C57BL/6 *Fmr1* KO mice showed higher susceptibility between P15 and P47, while it is between P14 and P94 in FVB *Fmr1* KO mice (Musumeci et al., 2000, 2007; Yan et al., 2005). This is confirmed in our experiments where P45 C57BL/6 *Fmr1* KO mice showed higher susceptibility to AGS than P90 mice. Clinical investigations reported that seizures are found in a small but significant subpopulation of FXS patients during the infancy and usually disappear with maturation (Hagerman and Stafstrom, 2009; Gauthey et al., 2010). The presently reported findings and previous works in the FXS animal model suggest that high homology exists between *Fmr1* KO mice and the humans with FXS.

We also analyzed the neuronal networks participating in the seizure activity in *Fmr1* KO mice. In particular, we analyzed FosB/ΔFosB expression as an alternative to previous studies based on c-Fos immunoreactivity (Chen and Toth, 2001), another tool being used for many years to track acute changes in neuronal network activity. The analysis of FosB/ΔFosB expression presents several advantages, since c-Fos reliability has been questioned in cases of recurrent neuronal synchronization, like the one typical of epileptic animals (Mello et al., 1996). Moreover, the turnover of c-Fos is very rapid when compared to that of other markers, such as FosB and ΔFosB (Madsen et al., 2006), making c-Fos expression extremely short living. In contrast, thanks to their stability, products of the *fosB* gene family tend to accumulate in repeatedly activated neurons (Chen et al., 1997; Kelz and Nestler, 2000), being the ideal tool available now to investigate network activity in epileptic animals (Chen et al., 1997; Biagini et al., 2005; Madsen et al., 2006). As expected, the acoustic stimulus induced an increased labeling for FosB/ΔFosB in regions involved in processing auditory information, especially in the medial geniculate body and primary acoustic area, both in WT and *Fmr1* KO mice, indicating that acoustic pathways had been activated. These findings are consistent with those by Chen and Toth (2001), demonstrating increased c-Fos expression after acoustic stimulus in specific thalamic regions of the FXS genotype, such as the medial geniculate body, compared to stimulated WT mice. We also observed enhanced FosB/ΔFosB immunoreactivity in the medial geniculate body of *Fmr1* KO compared with WT stimulated mice. At variance, no differences between the two genotypes were present in FosB/ΔFosB expression in the primary acoustic area, a result that is also in agreement with c-Fos immunodetection (Chen and Toth, 2001).

Basal levels of FosB/ΔFosB immunoreactivity in the medial geniculate body and primary acoustic area were similar in both genotypes, but a different scenario emerged in the hippocampal formation, in which FosB/ΔFosB immunoreactivity was unexpectedly higher in unstimulated P45 *Fmr1* KO mice compared to age-matched WTs, suggesting that regions notoriously involved in cognitive and emotional physiological functions (Koe et al., 2009; Herry et al., 2010) were hyperactive in our animal model of FXS. These differences were previously unreported in experiments based on antibodies to c-Fos (Chen and Toth, 2001; Li et al., 2002). However, the hippocampus is responsive to acoustic stimuli (Miller and Freedman, 1995) and, as shown in other models, there is a good evidence that some hippocampal regions are strictly related to the formation of memory traces of acoustic information (Huang et al., 2010). The link among acoustic information, seizures and the hippocampus has also been confirmed by evaluating patients affected by TLE (Boutros et al., 2008). In TLE, the hippocampus is consistently involved in epileptic neuronal synchronization (Avoli et al., 2002), thus the enhanced immunopositivity for FosB/ΔFosB found in the CA1 region and in the subiculum of *Fmr1* KO mice not exposed to the acoustic test could be interpreted as caused by unobserved spontaneous seizures. Alternatively, differences in the response to environmental stimuli or to stress could be taken into account (Miller et al., 1999; Lauterborn, 2004). The CA1 subfield is involved in the termination of the stress response by regulating the neuroendocrine axis that controls corticosterone levels, a function that strictly integrates the effects of stress with memory processing (Joëls et al., 2009). This hippocampal region is especially sensitive to stressful stimuli and can be markedly altered by chronic stress exposure both during development (Biagini et al., 1998) and in the adulthood (Biagini et al., 1993), circumstances that result in the impairment of stress response termination. Even with slightly different results, most likely due to the different type and duration of stress and the different time after stress when tissue was collected, others also demonstrated that changes in *c-fos* gene expression induced by stress are greater in *Fmr1* KO compared to WT mice (Lauterborn, 2004). In addition, the increase in corticosterone levels is greater in FXS mice compared to WT subjected to the same type of stress (Lauterborn, 2004). In analogy to patients affected by FXS, *Fmr1* KO mice presented with a prolonged return to basal corticosterone levels in response to acute stress, which could be related to an altered hippocampal feedback regulation (Markham et al., 2006).

We have evaluated a second marker of neuronal cell activity by characterizing p-ERK1/2-immunopositive cells. ERK is member of the mammalian mitogen-activated protein kinase (MAPK) family of serine/threonine kinases and regulates a variety of physiological and pathophysiological cellular activities (Subramaniam et al., 2004). This is a marker alternative to FosB. ERK1/2 phosphorylation is known to be followed by c-Fos activation. c-Fos phosphorylation is catalyzed by ERK1/2 at Ser374 and, furthermore, the further downstream reaction at Ser362 stabilizes c-Fos for several hours (Okazaki and Sagata, 1995; Murphy et al., 2002). No regulatory effects on FosB are instead reported by ERK1/2 activation (Miller et al., 1998). Accordingly, transactivation by c-Fos is mediated by two C-terminal motifs named HOB1 and HOB2, of which HOB1, absent in FosB (Herdegen and Leah, 1998), can be phosphorylated by ERK1/2. Consistently, our findings on FosB and p-ERK1/2 co-labeling demonstrate only a partial superimposition of these markers, suggesting that activated neurons were indeed responsive to different molecular pathways. It has been proposed a critical role for ERK1/2 in regulating social behaviors, and it has been suggested that it may be an important factor in human psychiatric disorders (Engel et al., 2009; Satoh et al., 2011). Interestingly, Hou et al. (2006) and Price et al. (2007) showed that the basal p-ERK1/2 levels in hippocampal synaptosomes were elevated in *Fmr1* KO mice compared to WTs, but p-ERK1/2 could not be increased by stimulating metabotropic glutamate receptor (mGluR) as instead in WT mice. There is, however, some controversy in the literature regarding ERK1/2 activation in *Fmr1* KO tissue (Hu et al., 2008; Gross et al., 2010) and given its critical role in synaptic plasticity, as well as in neurodevelopment and regulation of social behaviors, Wang and colleagues (2012) decided to investigate further this question using human brain tissue obtained from FXS patients. For the first time, they demonstrated that ERK1/2 phosphorylation is altered in neocortex and hippocampus of FXS patients, suggesting that there is a chronic activation of the MAPK/ERK kinase (MEK)-ERK1/2 pathway; in addition they confirmed these results in cortical tissue obtained from *Fmr1* KO mice (Wang et al., 2012). Similar upregulation of p-ERK1/2 in *Fmr1* KO mice was also found by Michalon et al. (2012). In line with these data, we observed a higher level of p-ERK1/2-immunopositive cells in basal conditions, but this difference was specifically found in the subiculum of *Fmr1* KO mice. This finding does not exclude that in fresh tissue, studied with different techniques (Michalon et al., 2012; Wang et al., 2012), enhanced ERK1/2 phosphorylation is found in other brain regions, but the subiculum could anyway represent an area of prominent ERK1/2 activation in the FXS model.

Activation of ERK1/2 by phosphorylation is strongly promoted by glutamate release during seizures and by stimulation of glutamate receptors (Jeon et al., 2000; Otani et al., 2003; Merlo et al., 2004; Houser et al., 2008). Interestingly, inhibition of ERK1/2 phosphorylation was shown to decrease *in vitro* ictogenesis induced by 4-aminopyridine (Merlo et al., 2004). Consistently with these data, a constitutively active form of MEK1 induced ERK1/2 activation and caused spontaneous epileptic seizures when conditionally expressed in the murine brain (Nateri et al., 2007). Recent experiments have cleared the relationship between ERK1/2 phosphorylation and the occurrence of AGS. Using the inhibitor of the MEK-ERK1/2 kinase cascade U0126, three different laboratories were able to block AGS in *Fmr1* KO mice (Osterweil et al., 2010; Michalon et al., 2012; Wang et al., 2012). To this regard, we found that p-ERK1/2 immunoreactivity is decreased in the subiculum, but not in perirhinal cortex, of *Fmr1* KO mice not manifesting AGS in response to acoustic stimulation. At variance, no significant changes of p-ERK1/2 immunoreactivity were observed in mice presenting with AGS induction. On the other hand, p-ERK1/2 levels were upregulated by the acoustic test in WT mice, in which we did not observe any epileptic response. This discrepancy suggests a differential regulation of ERK1/2 activity in normal and epilepsy-prone animals. Using pilocarpine to induce seizures, Houser and colleagues (2008) actually observed a decrease in p-ERK1/2 levels in naïve mice, whereas ERK1/2 phosphorylation increased immediately before the appearance of recurrent spontaneous seizures in the animals that experienced the pilocarpine-induced *status epilepticus*. Thus, it is possible that the enhanced phosphorylation of ERK1/2 observed in WT mice in the subiculum represents a molecular fingerprint of the exposure to subthreshold proconvulsive stimuli in presence of a normal genotype, whereas the increase of p-ERK1/2 basal levels found in *Fmr1* KO mice could be related to a proconvulsive genotype. In addition, an inefficient p-ERK1/2 dephosphorylation in the subiculum of *Fmr1* KO mice appears to be responsible for AGS induction, whereas enhanced phosphatase activity, acquired during maturation, could explain the resilience to proconvulsive stimuli in older *Fmr1* KO mice. This overall evidence suggests that abnormally elevated and timely maintained p-ERK1/2 levels in the subiculum are required for AGS induction in *Fmr1* KO mice. Notably, the changes we found in p-ERK1/2 levels were age-dependent, suggesting that this mechanism could be involved in the modification of seizure susceptibility during maturation in the FXS. Contrary to results obtained in the subiculum, no differences at all were observed in the perirhinal cortex of WT and *Fmr1* KO mice exposed to the test for AGS induction. This result is consistent with previous findings that showed defective neurotransmission in the subiculum of *Fmr1* KO mice (D'Antuono et al., 2003; Curia et al., 2009). In particular, p-ERK1/2 was shown to display regulatory properties on γ-aminobutyric acid (GABA) type A receptors by decreasing peak currents generated by α1 β2 γ2 combination of subunits, an effect prevented by UO126 treatment (Bell-Horner et al., 2006). This effect on GABA peak current could be particularly important in a background of decreased GABA tonic current, such as that found in *Fmr1* KO mice (Curia et al., 2009). The subiculum from patients affected by TLE, due to hippocampal sclerosis, was found to generate spontaneous epileptic activity *in vitro* (Cohen et al., 2002). Consistently, enhanced neuronal excitability was demonstrated in the subiculum of pilocarpine-treated rats, a model of TLE associated with brain damage, in which GABAergic neurons were significantly decreased (De Guzman et al., 2006). Thus, the present findings on markedly enhanced FosB/ΔFosB levels and p-ERK1/2 expression in the subiculum of *Fmr1* KO mice further support the view of a critical role of this limbic region in controlling the spread of seizure activity in neuronal networks also in models of genetic epilepsy.

### Conflict of interest statement

The authors declare that the research was conducted in the absence of any commercial or financial relationships that could be construed as a potential conflict of interest.
